# Supplementary material for: Intranasal delivery of pro-resolving lipid mediators rescues memory and gamma oscillation impairment in AppNL-G-F/NL-G-F mice
Source: Commun Biol. 2022 Mar 21;5:245. doi: 10.1038/s42003-022-03169-3 (PMC8938447; doi:10.1038/s42003-022-03169-3)
Supplement: Supplementary file 2 — Supplementary information [file 42003_2022_3169_MOESM2_ESM.pdf]

Supplementary Information

Supplementary Fig. 1a-b. Immunoreactivity for GFAP upon treatment of $\boldsymbol{App^{NL\text{-}G\text{-}F}}$ mice with pro-resolving lipid mediators. (a) Micrographs showing immunoreactivity for GFAP in WT mice and in $App^{NL\text{-}G\text{-}F}$ mice with and without treatment with pro-resolving lipid mediators (LMs). An increase in the labelled astrocytes can be seen in $App^{NL\text{-}G\text{-}F}$ mice compared to WT mice, both given vehicle. There is no clear difference in GFAP-positive astrocytes upon treatment of the $App^{NL\text{-}G\text{-}F}$ mice with LMs. **(b)** The area covered by GFAP immunoreactivity in the cerebral cortex and hippocampus was quantified by densitometry. Analysis of densitometry data shows increased levels in the $App^{NL\text{-}G\text{-}F}$ mice compared to WT mice in both brain regions, and no effect of treatment of the $App^{NL\text{-}G\text{-}F}$ mice with pro-resolving LMs. Mann-Whitney U test was used for comparisons between two groups and correction was performed manually by multiplying with number of comparisons. n = 5 mice/group, $^{*}P < 0.05$, $^{**}P < 0.01$, $^{***}P < 0.001$. Data are presented as mean ± S.E.M. Scale bars = 600 and 150 μm. GFAP = glial fibrillary acidic protein, WT = wild-type

Supplementary Fig. 2. Western blots of receptors and synaptic markers. Analysis of receptors for pro-resolving lipid mediators (LMs) (BLT1, ChemR23, LGR6, GPR18, and FPR2), glutamate and GABA receptors (GluR1, GluR4, $GABA_A1\alpha$), a synaptic marker (PSD95), and an inflammation marker (TREM2), was performed by Western blot in cortex and hippocampus of WT + Veh, $App^{NL\text{-}G\text{-}F}$ + Veh and $App^{NL\text{-}G\text{-}F}$ + LMs mice. Blots correspond to those shown in Fig. 4.

Supplementary Fig. 3. Brain cytokine and chemokine levels upon treatment of $\boldsymbol{App^{NL\text{-}G\text{-}F}}$ mice with pro-resolving lipid mediators. Homogenates of the cerebral cortex and hippocampus from WT mice and from $App^{NL\text{-}G\text{-}F}$ mice with and without treatment with pro-resolving lipid mediators (LMs) were analysed by Meso Scale V-plex immunoassay. The data in are expressed pg/ml. Statistical analysis was performed with Kruskal-Wallis test with Dunn's multiple comparisons *post hoc* test. n = 5 mice/group, $^{*}P < 0.05$. Data are presented as mean ±

S.E.M. IFN-γ = interferon-γ, IL = interleukin, IP-10 = interferon-γ-induced protein 10, KC-GRO = keratinocyte chemoattractant/human growth-regulated oncogene, MCP-1 = monocyte chemoattractant protein, MIP = macrophage inflammatory protein, TNF-α = tumour necrosis factor-α

Supplementary Fig. 4. Effects of pro-resolving lipid mediators on endogenous lipids in the brain of *App$^{NL\text{-}G\text{-}F}$* *mice*. Lipid extracts of the cerebral cortex and hippocampus from WT mice and from *App$^{NL\text{-}G\text{-}F}$* mice with and without treatment with pro-resolving lipid mediators (LMs) were analysed by LC-MS/MS. Kruskal-Wallis with Dunn's *post hoc* test was used for multiple comparisons. n = 5 mice/group. Data are presented as mean ± S.E.M. AA = arachidonic acid, DHA = docosahexaenoic acid, EPA = eicosapentaenoic acid, HDHA = hydroxy-docosahexaenoic acid, HETE = hydroxyeicosatetraenoic acid, LC-MS/MS = liquid chromatography/tandem mass spectrography, LXA_4 = lipoxin A_4, NPD1 = neuroprotectin D1, PG = prostaglandin, Rv = resolvin, WT = wild-type

Supplementary Fig. 5. Detection of deuterium-labelled lipid mediators in the brain of *App$^{NL\text{-}G\text{-}F}$* mice. Lipid extracts of the cerebral cortex (Cx) and hippocampus (Hippo) from two *App$^{NL\text{-}G\text{-}F}$* mice treated with deuterium-labelled pro-resolving lipid mediators (LMs) were analysed by LC-MS/MS. Small amounts of maresin 1 (MaR1)-d5, resolvin D1 (RvD1)-d5, RvD2-d5 and RvE1-d5 were detected. Data depict the ratio of these LMs to the internal control standard prostaglandin D2 (PGD2)-d4 for Cx and Hippo.

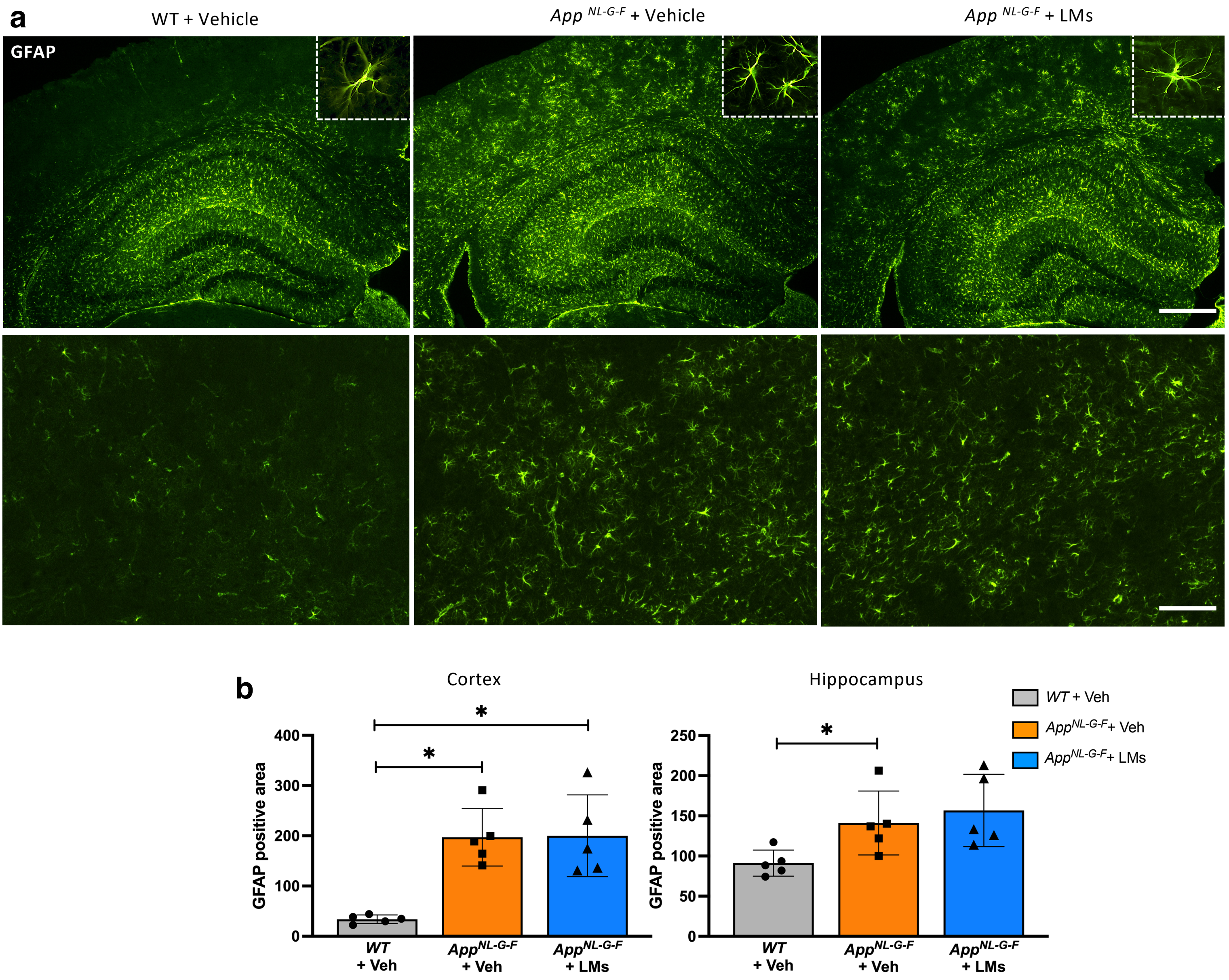

SUPPLEMENTARY FIGURE 1

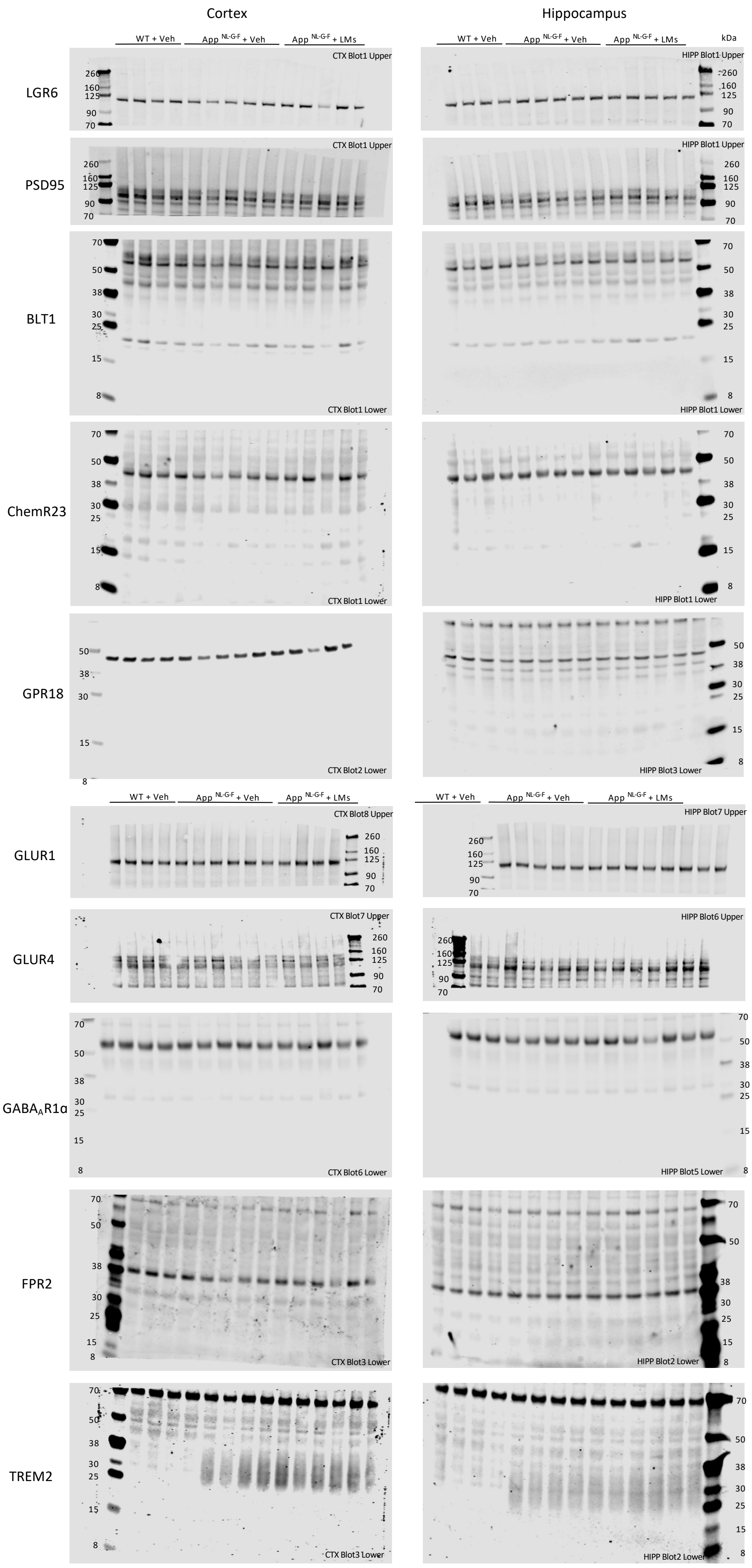

SUPPLEMENTARY FIGURE 2

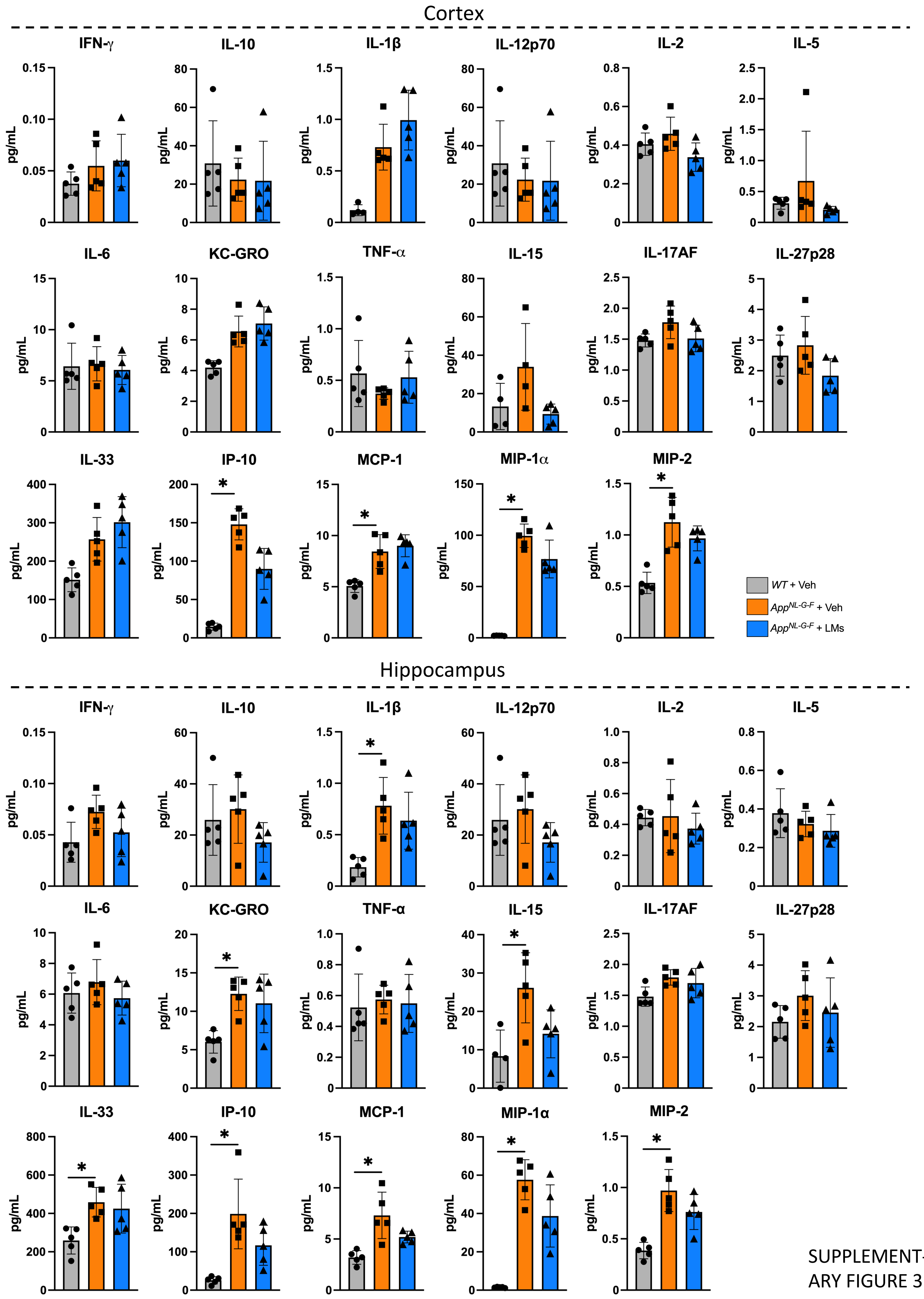

SUPPLEMENT-
ARY FIGURE 3

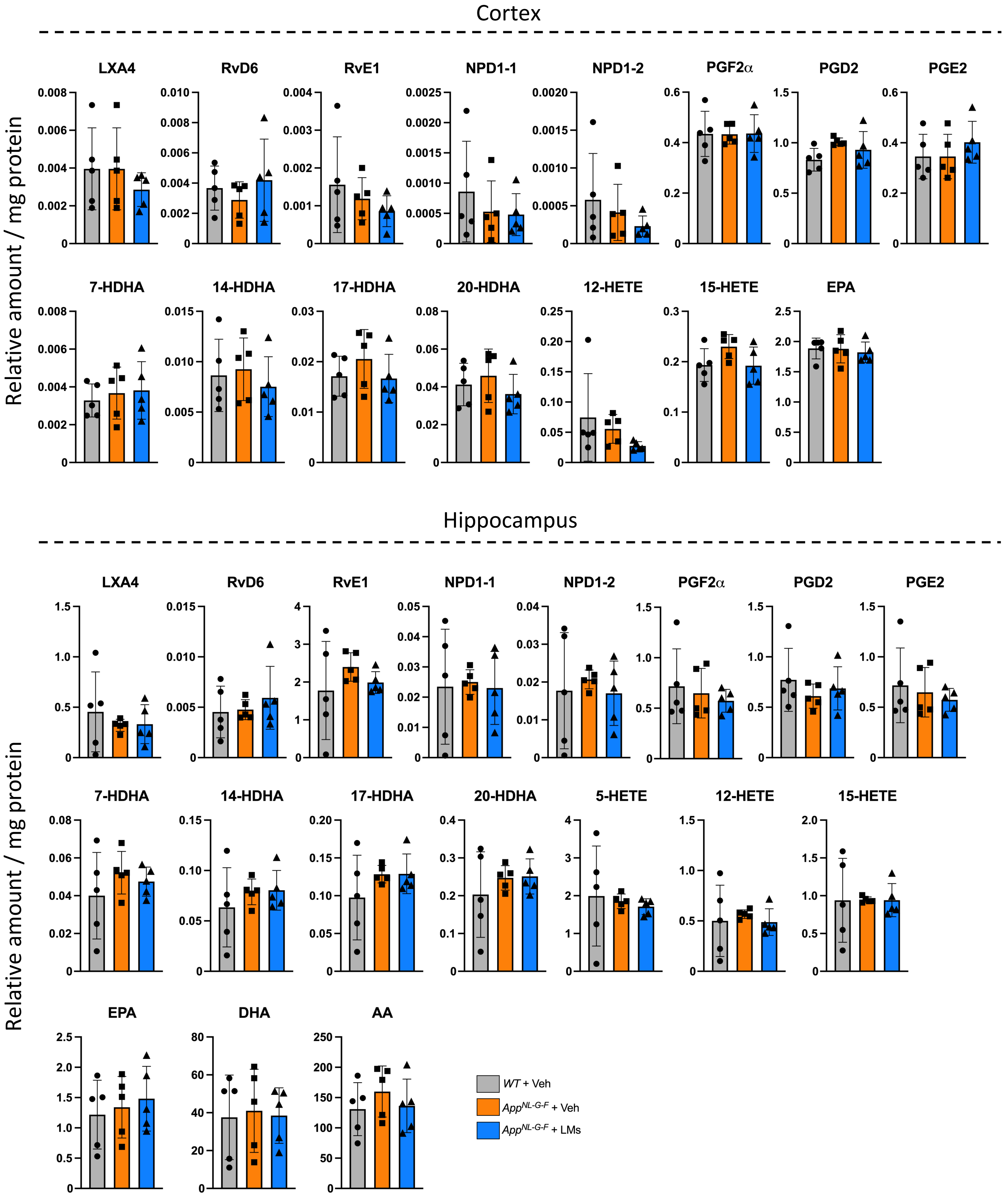

SUPPLEMENTARY FIGURE 4

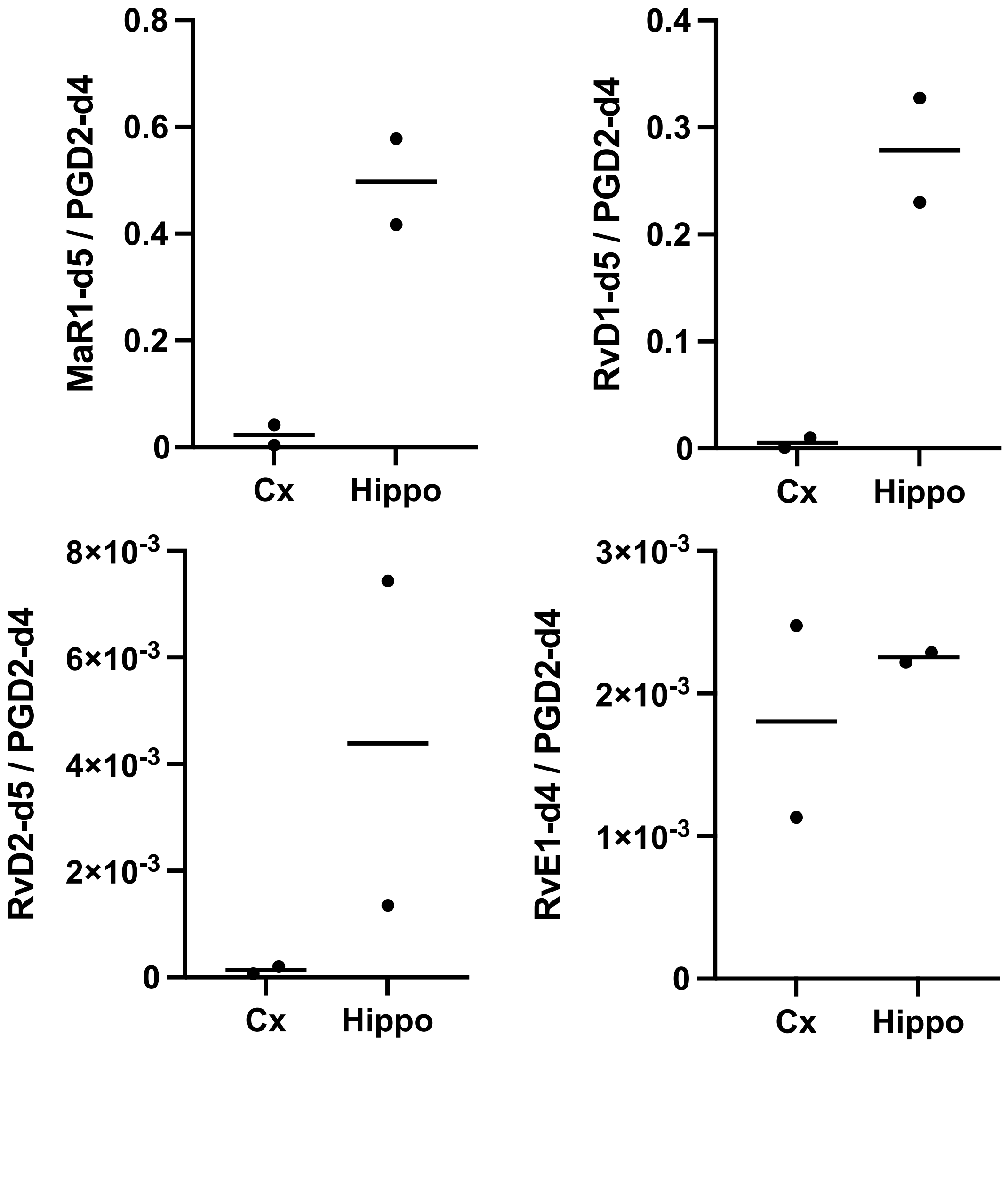

SUPPLEMENTARY FIGURE 5
