# Supplementary material for: Intranasal delivery of pro-resolving lipid mediators rescues memory and gamma oscillation impairment in AppNL-G-F/NL-G-F mice
Source: Commun Biol. 2022 Mar 21;5:245. doi: 10.1038/s42003-022-03169-3 (PMC8938447; doi:10.1038/s42003-022-03169-3)
Supplement: Supplementary file 3 — Description of Additional Supplementary Files [file 42003_2022_3169_MOESM3_ESM.pdf]

Description of Additional Supplementary Files

File name: Supplementary Data 1

Description: Source data for Figs 1-4, 6, and Supplementary Figs 1-4.
